# Online real-time learning of dynamical systems from noisy streaming data

**DOI:** 10.1038/s41598-023-49045-w

**Published:** 2023-12-19

**Authors:** S. Sinha, S. P. Nandanoori, D. A. Barajas-Solano

**Affiliations:** Pacific Northwest National Laboratory, Richland, WA 99354 USA

**Keywords:** Engineering, Electrical and electronic engineering

## Abstract

Recent advancements in sensing and communication facilitate obtaining high-frequency real-time data from various physical systems like power networks, climate systems, biological networks, etc. However, since the data are recorded by physical sensors, it is natural that the obtained data is corrupted by measurement noise. In this paper, we present a novel algorithm for online real-time learning of dynamical systems from noisy time-series data, which employs the Robust Koopman operator framework to mitigate the effect of measurement noise. The proposed algorithm has three main advantages: (a) it allows for online real-time monitoring of a dynamical system; (b) it obtains a linear representation of the underlying dynamical system, thus enabling the user to use linear systems theory for analysis and control of the system; (c) it is computationally fast and less intensive than the popular extended dynamic mode decomposition (EDMD) algorithm. We illustrate the efficiency of the proposed algorithm by applying it to identify the Van der Pol oscillator, the chaotic attractor of the Henon map, the IEEE 68 bus system, and a ring network of Van der Pol oscillators.

## Introduction

The field of dynamical systems was born with the works of Sir Issac Newton^1^. Since its humble beginnings, it has developed into a matured branch of pure mathematics with applications to most branches of science and engineering. For almost three hundred years, the approach to studying dynamical systems was model-based, in which the system under study is modeled from first principles. However, this approach has severe limitations when studying systems like power networks, biological networks, stock markets, etc., which are inherently highly nonlinear and typically large-scale. As such it is almost impossible to model these dynamical systems from first principles. The way to circumvent this problem is to use data-driven techniques, in which the governing equations of motion are learned directly from time-series data of the states of the system.

Of the various data-driven learning methods, in recent years, the Koopman operator approach^2–9^ has been used extensively for the data-driven learning of dynamical systems. Compared to machine learning (ML) methods, the Koopman operator framework has multiple advantages, with the major factor being the fact that the amount of training data required by Koopman operator approaches is far less compared to ML methods. Furthermore, the Koopman operator is a linear operator that generates an equivalent linear representation of the nonlinear system, which allows us to use concepts from linear systems theory for the analysis and control design for nonlinear systems^10,11^.

Given a dynamical system, instead of looking at the evolution of trajectories, the Koopman operator governs the evolution of functions on the state space under the dynamical map. The main advantage of this framework, as mentioned earlier, is the fact that the Koopman operator is a linear operator, but the trade-off is that it is an infinite-dimensional operator. Hence, given any general nonlinear system, the corresponding Koopman operator generates an infinite-dimensional linear system, which is an exact representation of the nonlinear system and, unlike linearization, is valid globally on the state space. Furthermore, the Koopman framework facilitates data-driven learning of dynamical systems^8,12^ and has been extensively used for data-driven learning in synthetic biology^13,14^, building systems^15,16^, climate systems^17^, robotics^18,19^, neuroscience^20^, and power systems^21–23^, where a finite-dimensional approximation of the Koopman operator is computed from time-series data of the dynamical states.

However, when it comes to using data-driven learning of real physical systems, it is often a requirement that the learning framework learns the dynamics in an online fashion and in real-time. This is especially relevant for power networks, stock markets, building systems etc., which demand constant monitoring to ensure efficient and reliable operation. For example, a power network is exposed to multiple adversarial events like sudden load changes, faults and attacks^24^ and as such, it is imperative to monitor the power network constantly to identify any adversarial event as soon as possible and to take appropriate measures to ensure safe operation. Therefore, there is a need for a framework that can learn the underlying dynamics online and in real time^25^. Moreover, since the data is obtained from physical sensors, it is often the case that the data obtained is corrupted by noise.

Koopman operator based learning of dynamical systems is one of the many ways to learn a dynamical system and recently, machine learning (ML) and deep learning (DL) based methods have gained attention. One of the popular methods is reservoir computing (RC)^26^ and in^27^ the authors have connected reservoir computing with Koopman operators, but the connection is only to dynamic mode decomposition (DMD). DMD is a very special case of computing the Koopman operator, where the dictionary functions are just the states of the system. Though DMD does well in some cases, it does not identify the attractor sets, which extended dynamic mode decomposition (EDMD) can do^4^ EDMD uses nonlinear dictionary functions and thus is more general than DMD and is known to capture the nonlinear characteristics of a systems much better than DMD^4^. Furthermore, in^28^, the authors propose Next Generation RC (NG-RC) which has been shown to use less data for training. However, in^29^ the authors show that NG-RC “frameworks struggle to learn the dynamics unless key information about the underlying system is already known”. Hence, on one hand the traditional RC require lot of data for training, whereas NG-RC needs some a priori information about the underlying dynamics for learning the system efficiently. Also, there is the issue of interpretability, which is not present in the Koopman framework.

In this paper, we aim to address the problem of data-driven learning from noisy data in an online fashion and in real-time. In particular, we use the Robust Koopman operator framework^6,30^ to mitigate the effect of measurement noise. and extend this work to provide a framework for computing the Koopman operator from similar streaming noisy data and show how the Koopman operator can be computed in an iterative manner. We further give evidence that the current framework is computationally more efficient compared to existing Koopman computation method, namely the extended dynamic mode decomposition (EDMD) algorithm. Furthermore, we propose a recursive algorithm to learn the Koopman representation of the underlying system, so that the Koopman operator gets updated as each new data point streams in. Thus the main contributions of the paper are the following:

- We propose a framework for computing the Koopman operator when there is both process and measurement noise.
- We propose an algorithm which can compute the Koopman operator in an online way and thus it facilitates real-time learning of the underlying dynamical system.
- We provide numerical evidence that the proposed algorithm is significantly faster than the existing extended dyanmic mode decomposition (EDMD) algorithm.

This manuscript is organized as follows: in “Transfer operators”, we discuss the basics of transfer operators for dynamical systems, followed by the formulation of the Robust Koopman operator computation algorithm in “Robust Koopman operator”. The main results of the paper are presented in “Online learning of robust Koopman operator”, where we derive and describe the online learning algorithm. In “Simulation results”, we illustrate the efficiency of the proposed algorithm by applying it to three different dynamical systems, namely, a Van der Pol oscillator, the IEEE 68 bus system and a ring network of Van der Pol oscillators. Finally, we conclude the paper in “Conclusions”.

## Transfer operators

Consider a discrete-time dynamical system

1 $$\begin{aligned} x_{t+1} = T(x_t) \end{aligned}$$

where $T:X\subset {\mathbb {R}}^N \rightarrow Z$ is assumed to be at least of class ${{\mathcal {C}}}^1$. Associated with the dynamical system (1) is the Borel-$\sigma$ algebra ${{\mathcal {B}}}(X)$ on *X* and the vector space ${{\mathcal {M}}}(X)$ of bounded complex-valued measures on *X*. With this, two linear operators, namely, the Perron–Frobenius (P–F) and the Koopman operator, can be defined as follows^2^:

### Definition 1

(*Perron–Frobenius operator*) The Perron–Frobenius operator ${\mathbb {P}}:{{\mathcal {M}}}(X)\rightarrow {\mathcal {M}}(X)$ is given by

$$\begin{aligned} {[}{\mathbb {P}}\mu {]}(A)=\int _{{X} }\delta _{T(x)}(A)d\mu (x)=\mu (T^{-1}(A)),\end{aligned}$$

where $\delta _{T(x)}(A)$ is the stochastic transition function that measures the probability that point *x* will reach the set *A* in one-time step under the system mapping *T*.

### Definition 2

(*Invariant measures*) Invariant measures are the fixed points of the P–F operator ${\mathbb {P}}$ that are also probability measures. Let ${\bar{\mu }}$ be the invariant measure then, $\bar{\mu }$ satisfies

$$\begin{aligned}{\mathbb {P}}\bar{\mu }=\bar{\mu }.\end{aligned}$$

If the state space *Z* is compact, it is known that the P–F operator admits at least one invariant measure.

### Definition 3

(*Koopman operator*) Given any $h\in {\mathcal {F}}$, ${\mathbb {U}}:{{\mathcal {F}}}\rightarrow {{\mathcal {F}}}$ is defined by

$$\begin{aligned}{[}{\mathbb {U}} h{]}(x)=h(T(x))\end{aligned}$$

where ${\mathcal {F}}$ is the space of function (observables) invariant under the action of the Koopman operator.

**Figure 1:**
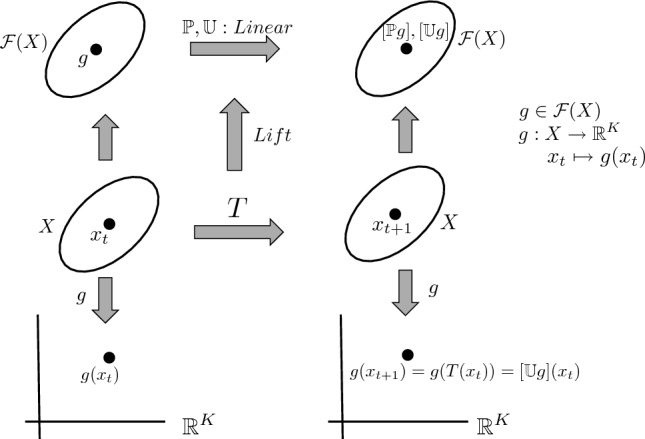
Perron–Frobenius and Koopman operators corresponding to a dynamical system.

Both the P–F and the Koopman operators are linear operators even if the underlying system is nonlinear and while the analysis is made tractable by linearity, the trade-off is that these operators are typically infinite-dimensional (see Fig. 1). In particular, the P–F and Koopman operators lift a dynamical system from a finite-dimensional space to generate an infinite-dimensional linear system on the space of distributions and functions respectively.

## Robust Koopman operator

The Koopman operator is infinite-dimensional, but for computational purposes, one has to compute a finite-dimensional approximation of the Koopman operator from time-series data. The most commonly used approach for computing the finite-dimensional approximation is known as extended dynamic mode decomposition (EDMD)^4^.

### Extended dynamic mode decomposition

Let

$$\begin{aligned}{[}x_1,x_2, \ldots , x_M{]}\in {\mathbb {R}}^{N\times M}\end{aligned}$$

be *M* data points from a *N*-dimensional system $x_t\mapsto T(x_t)$, which evolves on ${\mathbb {R}}^N$. Let

$$\begin{aligned}{\varvec{\Psi }}(x)=[\psi _1(x), \psi _2(x), \ldots , \psi _K(x)]:{\mathbb {R}}^N\rightarrow {\mathbb {R}}^K\end{aligned}$$

be the set of vector-valued Koopman observables such that each $\psi _i:{\mathbb {R}}^N\rightarrow {\mathbb {R}}$ is square-integrable. Let

2 $$\begin{aligned} \textbf{G} = \frac{1}{M}\sum _{i=1}^{M-1}{\varvec{\Psi }}(x_i)^\top \mathbf{\Psi }(x_i); \quad \textbf{A} = \frac{1}{M}\sum _{i=1}^{M-1}\mathbf{\Psi }(x_i)^\top {\varvec{\Psi }}(x_{i+1}). \end{aligned}$$

Then the finite-dimensional approximation $\textbf{K}\in {\mathbb {R}}^{K\times K}$ is obtained as the solution of the optimization problem

3 $$\begin{aligned} \min _\textbf{K}\parallel \textbf{G}{} \textbf{K}-\textbf{A}\parallel , \end{aligned}$$

where $\textbf{G}$ and $\textbf{A}$ are given by (2).

### Robust Koopman operator

The standard approach to compute the Koopman operator from time-series data is by solving the optimization problem (3). However, in presence of noise in the data, (3) leads to erroneous Koopman operators and one has to explicitly take into account the noise in the data, while computing the Koopman operator.

Consider a discrete time random dynamical system perturbed with additive noise so that the equations of motion are given by

4 $$\begin{aligned} x_{t+1}=F(x_t)+\xi _t=:T(x_t,\xi _t) \end{aligned}$$

where $T:X\times W \rightarrow X$ with $X\subset {\mathbb {R}}^N$ is assumed to be invertible with respect to *x* for each fixed value of $\xi$ and smooth diffeomorphism. We assume $\xi _t\in W$ is independent identically distributed (i.i.d) random variable with probability distribution $\vartheta$ i.e.,

$$\begin{aligned}\textrm{Prob}(\xi _t\in B)=\vartheta (B)\end{aligned}$$

for every set $B\subset W$ and all *t*. Let ${{\mathcal {B}}}(X)$ denote the Borel-$\sigma$ algebra on *X* and ${{\mathcal {M}}}(X)$ the vector space of bounded real-valued measure on *X*. With this, we consider snapshots of data set obtained from simulating a discrete time random dynamical system $x\rightarrow T(x,\xi )$ or from an experiment

5 $$\begin{aligned} X = [x_0,x_2,\ldots ,x_M] \end{aligned}$$

where $x_i\in X\subset {\mathbb {R}}^n$. The data-set $\{x_k\}$ is a sample path trajectory generated by a random dynamical system and could be corrupted by either process or measurement noise or both. Let $\{x_k\}_{k=1}^M$ be the one realization of the sample path trajectory generated by RDS.

#### Assumption 4

Let $\{x_k\}_{k=1}^M$ be the sample path trajectory of the RDS (4). We assume that the other sample path trajectories, $\{{\tilde{x}}_k\}_{k=1}^M$ satisfies

6 $$\begin{aligned} {\tilde{x}}_k=x_k+\delta _k, \;\;\;\delta _k\in \Delta \end{aligned}$$

for some norm bounded uncertainty set $\Delta$.

Note that the uncertainty set $\Delta$ will be related to set *W* containing the random variable $\xi$ and the probability distribution $\vartheta$. However, in our proposed deterministic approach to stochastic uncertainty, we assume that the uncertainty set $\Delta$ is norm-bounded. For example

7 $$\begin{aligned} \Delta :=\{\delta \in {\mathbb {R}}^n:\;\; \parallel \delta \parallel _2\le \rho \} \end{aligned}$$

restrict the 2-norm of $\delta$ to $\rho$. There can be other forms for the uncertainty set $\Delta$. For details see^6^.

We also consider measurement noise so that the measurements are of the form

8 $$\begin{aligned} x^m(t) = \tilde{x}(t) + \xi (t) \end{aligned}$$

where $\xi (t)$ is independent and normally distributed with zero mean and variance $\sigma ^2$.

The uncertainty in the data acts as an adversary which tries to maximize the residual error of the optimization problem which computes the Koopman operator, and hence to obtain the Koopman operator $\textbf{K}$ for uncertain data, a robust optimization problem is formulated as the following $\min -\max$ optimization problem^6,30^.

9 $$\begin{aligned} \min \limits _\textbf{K}\max _{\delta x_1,\ldots , \delta x_M\in \Delta }\parallel \textbf{G}_\delta \textbf{K}-\textbf{A}_\delta \parallel _F=:\min \limits _\textbf{K}\max _{\delta \in \Delta } {{\mathcal {J}}}(\textbf{K}, \textbf{G}_\delta ,\textbf{A}_\delta ) \end{aligned}$$

where

10 $$\begin{aligned}{} & {} \textbf{G}_\delta =\frac{1}{M}\sum _{i=1}^{M-1} \varvec{\Psi }({x}_i)^\top \varvec{\Psi }({x}_i+\delta x_i)\nonumber \\{} & {} \textbf{A}_\delta =\frac{1}{M}\sum _{i=1}^{M-1} \varvec{\Psi }({x}_i)^\top \varvec{\Psi }({x}_{i+1}+\delta x_{i+1}), \end{aligned}$$

with $\textbf{K},\textbf{G}_\delta ,\textbf{A}_\delta \in {\mathbb {C}}^{K\times K}$ and $\Delta$ is the bounded uncertainty set.

The robust optimization problem (9), is in general non-convex because the cost ${\mathcal {J}}$ may not be a convex function of $\delta$.

#### Proposition 5

*The optimization problem* (9) *can be approximated as*

11 $$\begin{aligned} \min \limits _\textbf{K}\max _{\delta \textbf{G},\delta \textbf{A}\in {\mathcal {U}}}\parallel (\textbf{G}+\delta \textbf{G})\textbf{K}-(\textbf{A}+\delta \textbf{A})\parallel _F \end{aligned}$$

*where* ${\mathcal {U}}$ *is a compact set in* ${\mathbb {R}}^{K\times K}$.

#### *Proof*

From Taylor series expansion we have, ${\varvec{\Psi }}(x_i+\delta x_i) = {\varvec{\Psi }}(x_i) + {\varvec{\Psi }}'(x_i) \delta x_i+h.o.t.$, where $\mathbf{\Psi }'(x_i)$ is the first derivative of ${\varvec{\Psi }}(x)$ at $x_i$. Hence,

$$\begin{aligned} \textbf{G}_{\delta }\approx & {}\, \textbf{G} + \frac{1}{M}\sum _{i=1}^{M-1}{\varvec{\Psi }}^\top (z_i)\delta z_i {\varvec{\Psi }}'(z_i)\\= &\, {} \textbf{G} +\delta \textbf{G} \end{aligned}$$

where $$\delta \textbf{G} = \frac{1}{M}\sum _{i=1}^{M}{\varvec{\Psi }}^\top (x_i)\delta z_i {\varvec{\Psi }}'(x_i)$$.

Moreover,

$$\begin{aligned} \parallel \delta \textbf{G} \parallel _F{} & {} = \parallel \frac{1}{M}\sum _{i=1}^{M}{\varvec{\Psi }}^\top (x_i)\delta x_i {\varvec{\Psi }}'(x_i) \parallel _F\\{} & {} \le \frac{1}{M}\sum _{i = 1}^{M}\parallel {\varvec{\Psi }}^\top (x_i)\delta x_i {\varvec{\Psi }}'(x_i)\parallel _F \\{} & {} \le \frac{1}{M}\sum _{i=1}^{M}\parallel {\varvec{\Psi }}^\top (x_i)\parallel _F \cdot \parallel \delta x_i \parallel _F \cdot \parallel {\varvec{\Psi }}'(z_i)\parallel _F \end{aligned}$$

Hence, $\delta \textbf{G}$ belongs to a compact set ${{\mathcal {U}}}_1$. Similarly, one can show $\textbf{A}_\delta \approx \textbf{A} + \delta \textbf{A}$ and $\delta \textbf{A}$ belongs to a compact set ${{\mathcal {U}}}_2$. Letting ${{\mathcal {U}}}={{\mathcal {U}}}_1\cup {{\mathcal {U}}}_2$, proves the proposition. $\square$

With this, we have the following theorem, which derives a regularized least-squares optimization problem whose solution is the Robust Koopman Operator.

#### Theorem 6

*The optimization problem*

12 $$\begin{aligned} \min \limits _\textbf{K}\max _{\delta \textbf{G},\delta \textbf{A}\in {\mathcal {U}}}\parallel (\textbf{G}+\delta \textbf{G})\textbf{K}-(\textbf{A}+\delta \textbf{A})\parallel _F \end{aligned}$$

*is equivalent to the following optimization problem*

13 $$\begin{aligned} \min \limits _\textbf{K}\parallel \textbf{G}{} \textbf{K}-\textbf{A}\parallel _F+\lambda \parallel \textbf{K}\parallel _F, \end{aligned}$$

*where* $\lambda >0$ *is a constant dependent on the bound of the noise*.

#### *Proof*

For a $K\times K$ matrix $M=[m_{i,j}]\in {\mathbb {R}}^{K\times K}$, let ${{\mathcal {M}}}$ denote the vector

$$\begin{aligned} {{\mathcal {M}}}=[m_{1,1},\ldots ,m_{K,1},m_{1,2},\ldots ,m_{K,2},\ldots , m_{K,K}]^\top . \end{aligned}$$

This follows from the fact that ${\mathbb {R}}^{K\times K} \cong {\mathbb {R}}^{K^2}$. Hence,

$$\begin{aligned}\parallel M\parallel _F = \parallel {{\mathcal {M}}}\parallel _2.\end{aligned}$$

For two matrices *A* and *B*, let $A \otimes B$ denote the Kronecker product of *A* and *B*. Let ${{\mathcal {K}}}$ be the vector form of $\textbf{K}$ and let ${\mathcal {A}}$ and $\delta {{\mathcal {A}}}$ be the vector forms of *A* and $\delta A$, respectively.

Then, the min–max optimization problem can be written as

14 $$\begin{aligned} {{\mathcal {J}}}= & {} \min _\textbf{K}\max _{\delta \textbf{G},\delta \textbf{A} \in {\mathcal {U}}}\parallel (\textbf{G}+\delta \textbf{G})\textbf{K}-(\textbf{A}+\delta \textbf{A})\parallel _F\nonumber \\ {}= & {} \min _{{\mathcal {K}}}\max _{\delta \textbf{G},\delta {{\mathcal {A}}}\in {{\mathcal {U}}}}\parallel [(\textbf{G}+\delta \textbf{G})\otimes I_K]{{\mathcal {K}}} - ({\mathcal {A}}+\delta {{\mathcal {A}}})\parallel _F\nonumber \\= & {} \min _{{\mathcal {K}}}\max _{\delta \textbf{G},\delta {{\mathcal {A}}}\in {\mathcal {U}}}\parallel [(\textbf{G}+\delta \textbf{G})\otimes I_K]{{\mathcal {K}}} - ({\mathcal {A}}+\delta {{\mathcal {A}}})\parallel _2 \end{aligned}$$

where $I_K$ is the $K\times K$ identity matrix. Writing $\textbf{G}\otimes I_K$ as $\hat{G}$ and $\delta \textbf{G}\otimes I_K$ as $\delta \hat{G}$, the optimization problem (14) can be written as

15 $$\begin{aligned} {{\mathcal {J}}} = \min _{{\mathcal {K}}}\max _{\begin{array}{c} \delta {\hat{G}}\in \Pi _K{{\mathcal {U}}}\\ \delta {{\mathcal {A}}}\in {{\mathcal {U}}} \end{array}}\parallel ({\hat{G}}+\delta {\hat{G}}){{\mathcal {K}}} - ({{\mathcal {A}}}+\delta {\mathcal {A}})\parallel _2, \end{aligned}$$

where $\Pi _K{{\mathcal {U}}}$ is the projection of ${\mathcal {U}}$ on ${\mathbb {R}}^{K\times K}\otimes {\mathbb {R}}^{K\times K}$.

Fix $\textbf{K}\in {\mathbb {R}}^{K\times K}$ and let

16 $$\begin{aligned} r = \max _{\begin{array}{c} \delta {\hat{G}}\in \Pi _K{{\mathcal {U}}}\\ \delta {\mathcal {A}}\in {{\mathcal {U}}} \end{array}}\parallel ({\hat{G}}+\delta {\hat{G}}){{\mathcal {K}}} - ({{\mathcal {A}}}+\delta {{\mathcal {A}}})\parallel _2 \end{aligned}$$

be the worst-case residual. Then,

17 $$\begin{aligned} \begin{aligned} r&\le \max _{\begin{array}{c} \delta {\hat{G}}\in \Pi _K\bar{\Delta }\\ \delta {{\mathcal {A}}}\in {{\mathcal {U}}} \end{array}}\parallel {{\hat{G}}}{{\mathcal {K}}} - {{\mathcal {A}}} \parallel _2 + \parallel \delta {{\hat{G}}}{{\mathcal {K}}} - \delta {{\mathcal {A}}} \parallel _2\le \parallel {{\hat{G}}}{{\mathcal {K}}} - {{\mathcal {A}}} \parallel _2 + \lambda \parallel {{\mathcal {K}}} - \mathbbm {1} \parallel _2\\&\le \parallel {{\hat{G}}}{{\mathcal {K}}} - {{\mathcal {A}}} \parallel _2 + \lambda \sqrt{\parallel {{\mathcal {K}}}\parallel _2^2 + K}= \parallel \textbf{G} \textbf{K} - \textbf{A} \parallel _F + \lambda \sqrt{\parallel \textbf{K}\parallel _F^2 + K} \end{aligned} \end{aligned}$$

Again, choose $[\delta \hat{G} \quad \delta \hat{A}]$ as

$$\begin{aligned}{[}\delta \hat{G} \quad \delta \hat{A}{]}= \frac{\lambda u}{\sqrt{\parallel {{\mathcal {K}}}\parallel _2^2 + K}}[{{\mathcal {K}}}^\top \quad K],\end{aligned}$$

where

18 $$\begin{aligned} u = {\left\{ \begin{array}{ll} \frac{\hat{G}{{\mathcal {K}}}-\hat{A}}{\parallel \hat{G}{{\mathcal {K}}}-\hat{A} \parallel }, \text { if } \hat{G}{{\mathcal {K}}}\ne \hat{A}\\ \text {any unit norm vector otherwise.} \end{array}\right. } \end{aligned}$$

Then,

19 $$\begin{aligned} \begin{aligned} r&= \max _{\begin{array}{c} \delta {\hat{G}}\in \Pi _K\bar{\Delta }\\ \delta \hat{A}\in {{\mathcal {U}}} \end{array}}\parallel ({\hat{G}}{{\mathcal {K}}}-\hat{A}) + (\delta {\hat{G}}{{\mathcal {K}}}-\delta \hat{A})\parallel _F\\&= \max _{\begin{array}{c} \delta {\hat{G}}\in \Pi _K\bar{\Delta }\\ \delta \hat{A}\in {{\mathcal {U}}} \end{array}} \left\| ({\hat{G}}{{\mathcal {K}}}-\hat{A}) + \lambda \left( \frac{\hat{G}{{\mathcal {K}}}-\hat{A}}{\parallel \hat{G}{{\mathcal {K}}}-\hat{A} \parallel }{{\mathcal {K}}}^\top {{\mathcal {K}}}+K\frac{\hat{G}{{\mathcal {K}}}-\hat{A}}{\parallel \hat{G}{{\mathcal {K}}}-\hat{A} \parallel } \right) \right\| _F\\&\ge \parallel ({\hat{G}}{{\mathcal {K}}}-\hat{A})\parallel _F +\lambda \left\| \left( \frac{\hat{G}{{\mathcal {K}}}-\hat{A}}{\parallel \hat{G}{{\mathcal {K}}}-\hat{A} \parallel }{{\mathcal {K}}}^\top {{\mathcal {K}}} +K\frac{\hat{G}{{\mathcal {K}}}-\hat{A}}{\parallel \hat{G}{{\mathcal {K}}}-\hat{A} \parallel } \right) \right\| _F \\&\ge \parallel ({\hat{G}}{{\mathcal {K}}}-\hat{A})\parallel _F + \lambda \sqrt{{{\mathcal {K}}}^\top {{\mathcal {K}}}+K}= \parallel \textbf{G} \textbf{K} - \textbf{A} \parallel _F + \lambda \sqrt{\parallel \textbf{K}\parallel _F^2 + K} \end{aligned} \end{aligned}$$

Hence, from (17) and (19), the worst case residual is

20 $$\begin{aligned} r = \min _{\textbf{K}}\parallel \textbf{G}{} \textbf{K} - \textbf{A} \parallel _F + \lambda \sqrt{\parallel \textbf{K}\parallel _F^2 +K}. \end{aligned}$$

Since, *K* is a constant, the $\textbf{K}$ that minimizes *r* in (20) is the same $\textbf{K}$ that minimizes

$$\begin{aligned}\parallel \textbf{G}{} \textbf{K} - \textbf{A} \parallel _F + \lambda \parallel \textbf{K}\parallel _F.\end{aligned}$$

$$\square $$

## Online learning of robust Koopman operator

The Koopman operator is generally computed by solving the optimization problem (13) or directly using the formula

21 $$\begin{aligned} \textbf{K}= (\textbf{G}+ \lambda I)^{-1} \textbf{A}, \end{aligned}$$

where *I* is the identity matrix of appropriate dimensions. Note that for computing the Koopman operator one needs to use the entire dataset and hence when a new data point streams in the Koopman operator needs to be recalculated from scratch using the new larger data set. However, this requires the inversion of a matrix, which is computationally expensive, especially for data-sets obtained from large-dimensional systems. This necessitates developing a recursive algorithm for Robust Koopman operator computation. In this section, we describe an algorithm that computes the Koopman operator recursively and thus reducing the computational cost.

Let

22 $$\begin{aligned} X_M = [x_1,x_2,\ldots ,x_M],&Y_M = [y_1,y_2,\ldots ,y_M] \end{aligned}$$

be *M* data points obtained from simulation of a dynamical system $x\mapsto T(x)$ or from an experiment, where $y_i=T(x_i)$. Let

23 $$\begin{aligned} \begin{aligned}{}&{\textbf{G}}_M={\varvec{\Psi }}(X_M)^\top {\varvec{\Psi }}(X_M)\\&{\textbf{A}}_M={\varvec{\Psi }}(X_M)^\top {\varvec{\Psi }}(Y_M) \end{aligned} \end{aligned}$$

be the data points in the lifted space $({\mathbb {R}}^K)$, where the points $x_i$ and $y_i$ are mapped by the Koopman dictionary functions ${\varvec{\Psi }}$. Let

24 $$\begin{aligned} \textbf{K}_M = ({{\textbf{G}}_{M}}+\lambda I)^{-1} {{\textbf{A}}_{M}} \end{aligned}$$

be the Koopman operator obtained by using the *M* data points. Now, a new data point $(x_{M+1},y_{M+1})$ is acquired. The problem is to update the Koopman operator ${\textbf{K}}_M$ to $\textbf{K}_{M+1}$, without explicitly computing the inverse of $({\textbf{G}}_{M+1}+\lambda I)$.

Note that (24) can be rewritten as

25 $$\begin{aligned} {\textbf{A}}_M = ({\textbf{G}}_{M}+\lambda I){\textbf{K}}_M = \hat{\textbf{G}}_M{\textbf{K}}_M. \end{aligned}$$

where

26 $$\begin{aligned} \begin{aligned} \hat{\textbf{G}}_M&= {\varvec{\Psi }}(X_M)^\top {\varvec{\Psi }}(X_M)+\lambda I\\&=\sum _{i=1}^M{\varvec{\Psi }}(X_i)^\top {\varvec{\Psi }}(X_i) + \lambda I\\ {\textbf{A}}_M&= {\varvec{\Psi }}(X_M)^\top {\varvec{\Psi }}(Y_M)=\sum _{i=1}^M\mathbf{\Psi }(X_i)^\top {\varvec{\Psi }}(Y_i) \end{aligned} \end{aligned}$$

and ${\varvec{\Psi }}(X_i)$ and ${\varvec{\Psi }}(Y_i)$ are the *i*th columns of ${\varvec{\Psi }}(X_M)$ and ${\varvec{\Psi }}(Y_M)$ respectively.

Now, as the new data point $y_{M+1}=T(x_{M+1})$ streams in, the updated Koopman operator ${\textbf{K}}_{M+1}$ satisfies

27 $$\begin{aligned} \hat{\textbf{G}}_{M+1}{\textbf{K}}_{M+1} = {\textbf{A}}_{M+1}, \end{aligned}$$

where

28 $$\begin{aligned} \begin{aligned}{}&\hat{\textbf{G}}_{M+1} = \sum _{i=1}^{M+1}{\varvec{\Psi }}(X_i)^\top {\varvec{\Psi }}(X_i) + \lambda I\\&{\textbf{A}}_{M+1}=\sum _{i=1}^{M+1}{\varvec{\Psi }}(X_i)^\top {\varvec{\Psi }}(Y_i). \end{aligned} \end{aligned}$$

Now,

$$\begin{aligned} \hat{\textbf{G}}_{M+1} =&\sum _{i=1}^{M+1}{\varvec{\Psi }}(X_i)^\top {\varvec{\Psi }}(X_i) + \lambda I\\ =&\left( \sum _{i=1}^{M}{\varvec{\Psi }}(X_i)^\top {\varvec{\Psi }}(X_i) + \lambda I\right) + {\varvec{\Psi }}(X_{M+1})^\top {\varvec{\Psi }}(X_{M+1})\\ =&\hat{\textbf{G}}_{M} + {\varvec{\Psi }}(X_{M+1})^\top {\varvec{\Psi }}(X_{M+1}). \end{aligned}$$

Hence, using the matrix inversion lemma, we have

29 $$\begin{aligned} \hat{\textbf{G}}_{M+1}^{-1} = \hat{\textbf{G}}_M^{-1} - \frac{\hat{\textbf{G}}_M^{-1}\mathbf{\Psi }(X_{M+1})^\top {\varvec{\Psi }}(X_{M+1})\hat{\textbf{G}}_M^{-1}}{1 + \mathbf{\Psi }(X_{M+1}) \hat{\textbf{G}}_M^{-1} {\varvec{\Psi }}(X_{M+1})^\top }. \end{aligned}$$

Moreover,

30 $$\begin{aligned} \nonumber {\textbf{A}}_{M+1}= & {} \sum _{i=1}^{M+1}{\varvec{\Psi }}(X_i)^\top {\varvec{\Psi }}(Y_i)\nonumber \\= & {} {\textbf{A}}_M + {\varvec{\Psi }}(X_{M+1})^\top {\varvec{\Psi }}(Y_{M+1}). \end{aligned}$$

Hence, from (27),

31 $$\begin{aligned} {\textbf{K}}_{M+1}= &\, {} \hat{\textbf{G}}_{M+1}^{-1}{\textbf{A}}_{M+1} \nonumber \\= & \,{} \left( \hat{\textbf{G}}_M^{-1} - \frac{\hat{\textbf{G}}_M^{-1}{\varvec{\Psi }}(X_{M+1})^\top {\varvec{\Psi }}(X_{M+1})\hat{\textbf{G}}_M^{-1}}{1 + {\varvec{\Psi }}(X_{M+1}) \hat{\textbf{G}}_M^{-1} {\varvec{\Psi }}(X_{M+1})^\top }\right) \end{aligned}$$

32 $$\begin{aligned}{} & {} \, \times \left( {\textbf{A}}_M + {\varvec{\Psi }}(X_{M+1})^\top {\varvec{\Psi }}(Y_{M+1})\right) . \end{aligned}$$

Equation (31) gives the formula for updating the Koopman operator as new data streams in, without explicitly computing the inverse at every step, thus reducing the computational cost and hence improving efficiency.

### Initialization of the algorithm

Equation (31) gives the updated Koopman ${\textbf{K}}_{M+1}$ operator in terms of quantities computed from the previous time step. Note that the updated Koopman operator ${\textbf{K}}_{M+1}$ depends on an inverse, namely, $\hat{\textbf{G}}_M^{-1}$. Hence, for computing the Koopman operator ${\textbf{K}}_1$, one needs to initialize both $\hat{\textbf{G}}_0$ and ${\textbf{A}}_0$. One potential way out of this situation is to compute the Koopman operator ${\textbf{K}}_q$ using the initial *q* data points $(x_i,y_i)$, $i=1,2,\ldots ,q$, $q<M$ as

$$\begin{aligned}{\textbf{K}}_q = \hat{\textbf{G}}_q^{-1}{\textbf{A}}_q\end{aligned}$$

and use the corresponding $\hat{\textbf{G}}_q$ and ${\textbf{A}}_q$ to compute the updated Koopman operators ${\textbf{K}}_n$, $n>q$.

For practical applications, we set

$$\begin{aligned}\hat{\textbf{G}}_0 = \delta I_K, \quad {\textbf{A}}_0 = 0_K,\end{aligned}$$

where $\lambda >0$, $I_K$ is the $K\times K$ identity matrix, and $0_K$ is the $K\times K$ zero matrix.

#### *Remark 7*

Initializing the parameter $\delta$ can be tricky and usually one should run the algorithm multiple times, with different $\delta$ on a given training dataset and choose the one which has the lowest error for some validation dataset. However, since the Koopman operator is a Markov operator one does not need to look at the error for many different runs, but can just choose the $\delta$ for which the Koopman operator has at least one unit eigenvalue.

**Algorithm 1:**
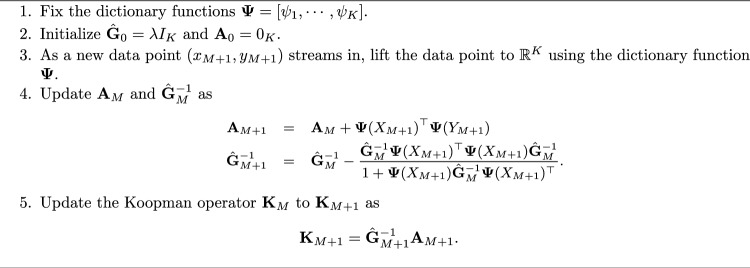
Algorithm for online Koopman Operator computation using streaming data.

## Simulation results

In this section, we demonstrate and discuss the properties of the proposed framework for iterative learning of dynamical models from noisy time-series data. All the simulations were done on a 2019 Macbook Pro with 16GB 2667 MHz DDR4 RAM and 2.3 GHz 8-Core Intel Core i9 processor.

### Van der Pol oscillator

**Figure 2:**
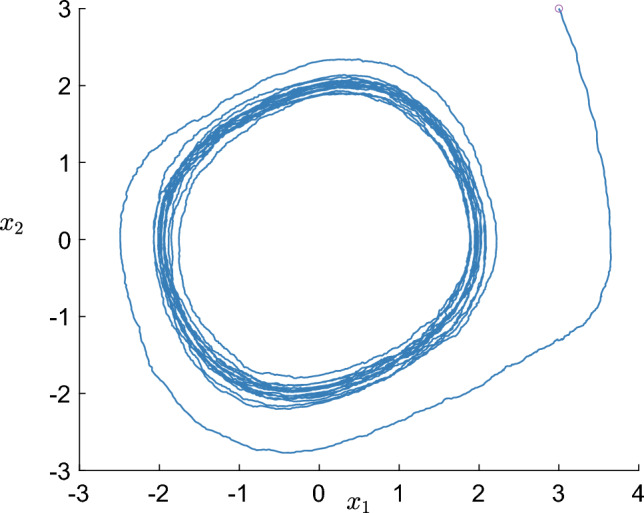
Phase portrait of noisy Van der Pol oscillator.

Consider a noisy Van der Pol oscillator, whose equation of motion is given by

$$\begin{aligned}\ddot{x}_t=\mu (1-x_t^2) - x_t + \sigma \xi _t,\end{aligned}$$

where $x\in {\mathbb {R}}$ is the position variable, $\mu \ge 0$ is the damping co-efficient, $\sigma >0$ is a constant, and $\xi _t\in {\mathbb {R}}^2$ is independent and identically distributed Gaussian noise of zero mean and unit variance. For simulation purposes, we chose $\mu = 0.8$ and $\sigma = 0.2$ and data were sampled at an interval of 0.01 s. The phase portrait of the noisy Van der Pol oscillator is shown in Fig. 2.

**Figure 3:**
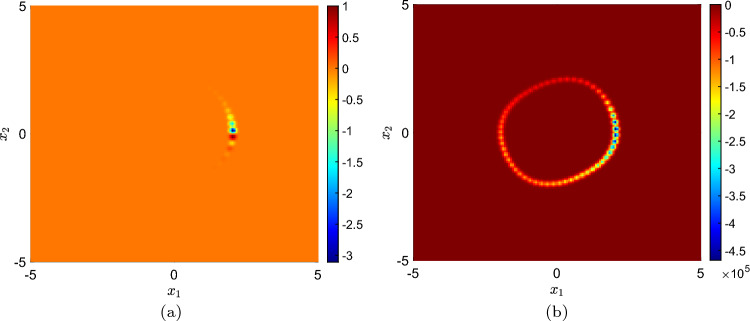
(**a**) The Koopman spectrum, computed from 500-time points data identifies the stable limit cycle of the Van der Pol oscillator partially. (**b**) After 2000 iterations, the Koopman spectra recover the entire stable limit cycle.

We considered a single trajectory data, from a random initial condition, as the training data and used 40 radial Gaussian basis functions as the Koopman observables. Figure 3 shows that the RR-EDMD (recursive robust extended dynamic mode decomposition) algorithm recovers the stable limit cycle of the Van der Pol oscillator as new data-points stream in and the Koopman operator gets updated. In particular, Fig. 3a shows that the eigenvector corresponding to the unit eigenvalue of the Koopman operator obtained after 500 data points have streamed in has partially identified the stable limit cycle. However, as more data points stream in, the identified region of the limit cycle grows and in Fig. 3b we find that with 2000 data points, the complete limit cycle has been identified.

**Figure 4:**
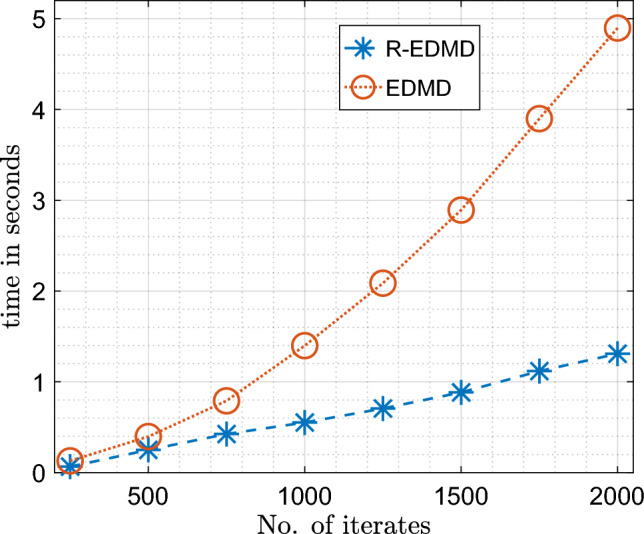
Comparison of computation time for recursive learning using normal EDMD and RR-EDMD.

However, the biggest advantage of the RR-EDMD formulation is the fact that it is much faster compared to EDMD. To compare the computational cost of RR-EDMD and EDMD, we present in Fig. 4 the computation times of both the algorithms for learning the Koopman operator as new data-points stream in. It can be seen the computation time of the RR-EDMD algorithm varies linearly with the number of iterates, whereas for EDMD it varies almost quadratically. This establishes a clear advantage of the RR-EDMD algorithm over EDMD and it is this fact that facilitates the use of RR-EDMD framework for online real-time learning of dynamical systems from streaming data.

### Henon map

In this example we consider a chaotic system, namely the Henon map. The Henon map is a two dimensional discrete-time system with a chaotic attractor with the equations of motion are given by

33 $$\begin{aligned} \begin{aligned}{}&x_{t+1} = 1 - ax_t^2 + y_t\\&y_{t+1} = bx_t. \end{aligned} \end{aligned}$$

**Figure 5:**
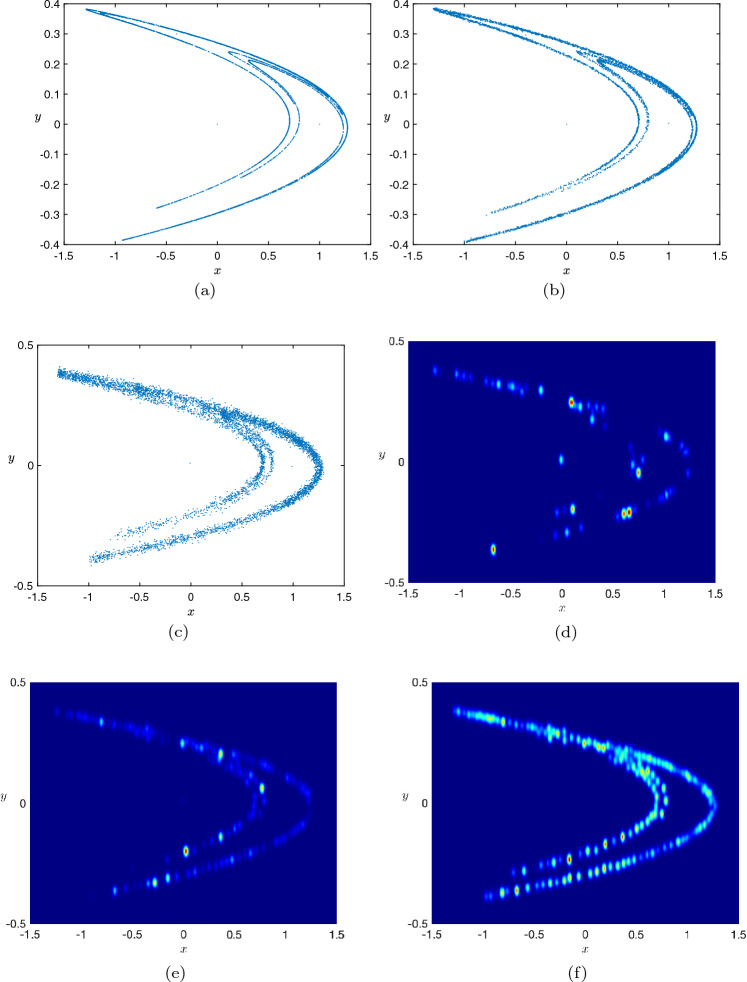
(**a**) Phase portrait of the deterministic Henon map. (**b**) Phase portrait of the Henon map with only process noise. (**c**) Phase portrait of the Henon map with both process and measurement noise. (**d**) Leading eigenfunction of the Koopman operator after 100 iterates. (**e**) Leading eigenfunction of the Koopman operator after 200 iterates. (**f**) Leading eigenfunction of the Koopman operator after 2000 iterates.

For this example, we added both process and measurement noise so that the noisy Henon map is given by

34 $$\begin{aligned} \begin{pmatrix} x_{t+1}\\ y_{t+1} \end{pmatrix} = \begin{pmatrix} 1 - ax_t^2 + y_t\\ bx_t \end{pmatrix} + \xi _t;\ \Theta _t = \begin{pmatrix} x_t\\ y_t \end{pmatrix} + \eta _t, \end{aligned}$$

where $\xi _t$ is the 2-dimensional i.i.d. Gaussian process noise with zero mean and variance 0.002, $\Theta _t$ is the observed output and $\eta _t$ is the 2-dimensional i.i.d. Gaussian measurement noise with zero mean and variance 0.01. Note that in order to keep the chaotic nature of the Henon map intact, we use very small process noise. With this the phase portrait for the deterministic Henon map, the phase portrait for Henon map with only process noise and the phase portrait for Henon map with both process and measurement noise state are shown in Fig. 5a–c.

With these the regularization parameter in Algorithm 1 was chosen to be $\lambda = 0.008$ and the initialization parameter $\delta$ was set to 0.001. As can be seen from Fig. 5d–f, our iterative framework gradually recovers the chaotic attractor from the noisy data. In particular, figure shows the leading eigenfunctions (eigenvector of the Koopman operator corresponding to the largest eigenvalue) on the phase space and we see that as more data points stream in, the chaotic attractor is indeed being identified.

### IEEE 68 bus system

**Figure 6:**
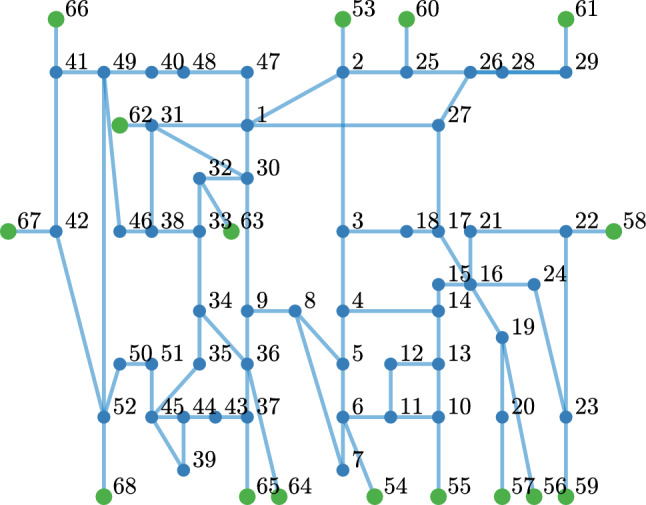
One line diagram representation of IEEE 68 bus network.

We now discuss the application of the proposed RR-EDMD algorithm to a power system. We consider synthetic time-series measurements corresponding to the 68-bus power network with 16 generators shown in Fig. 6. The synthetic measurements are generated using GridSTAGE^31^, a power system toolbox-based simulation platform that emulates PMU measurements corresponding to load changes or faults or adversarial actions.

**Figure 7:**
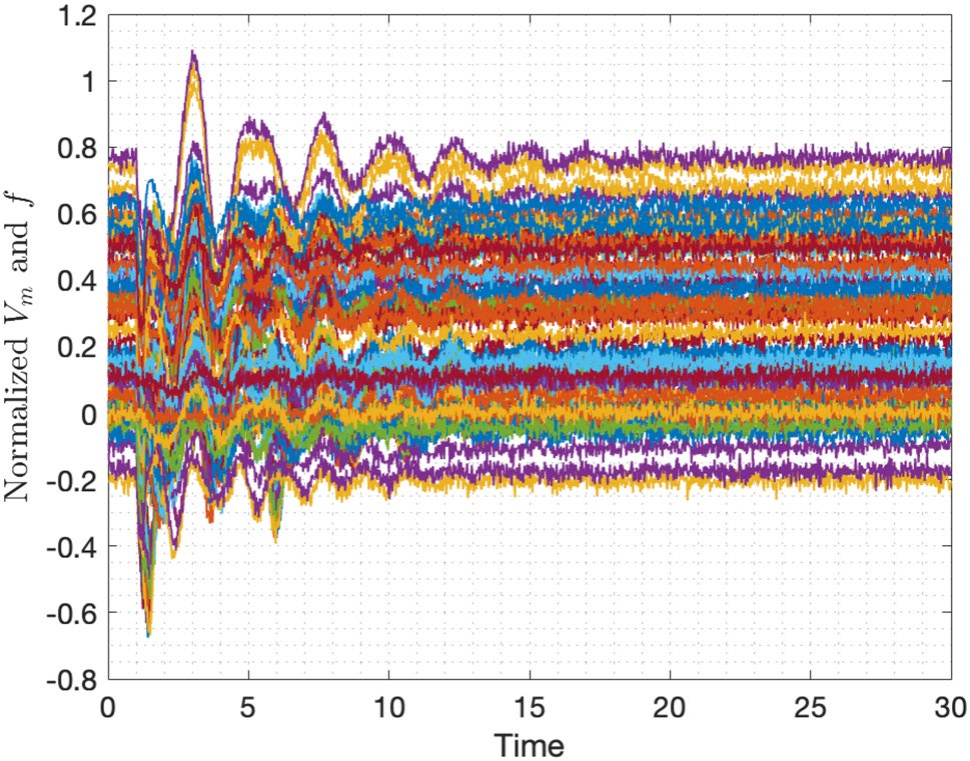
Noisy time-series data of frequency and voltage obtained from PMUs.

For the real-time learning of power system dynamics, random and large load changes are created using GridSTAGE. Although the synthetic time-series measurements obtained through GridSTAGE are free of noise, the practical PMU measurements will not be due to the effect of communication uncertainties. Hence, random noise is artificially added to the synthetic PMU measurements to mimic practical use cases and ensure the proper applicability of the proposed real-time learning framework. The noise-corrupted PMU measurements are shown in Fig. 7. Since the operating point of the underlying power network keeps changing due to random load changes or faults or due to the change in the operating conditions, the dynamics learnt from PMU data will not be valid for all operating conditions and need to be updated. Therefore, the proposed recursive learning method is particularly useful here for continuously learning the system dynamics from PMU data.

The PMU measurements record data at a rate of 40–80 measurements per second. In this study, we fix its rate to 50 measurements per second and each PMU measures several system states such as frequency, rate of change of frequency, voltage magnitude, voltage angle, current flow through the connected branches are available. Furthermore, to capture the noise in the real-time PMU measurements, we manually add the noise to the measurements such that the added noise is i.i.d. Gaussian with zero mean and variance 0.3. To demonstrate the proposed robust recursive Koopman operator to understand the system evolution from noisy data in real-time, we perform the analysis considering the important states, frequency and voltage magnitude.

**Figure 8:**
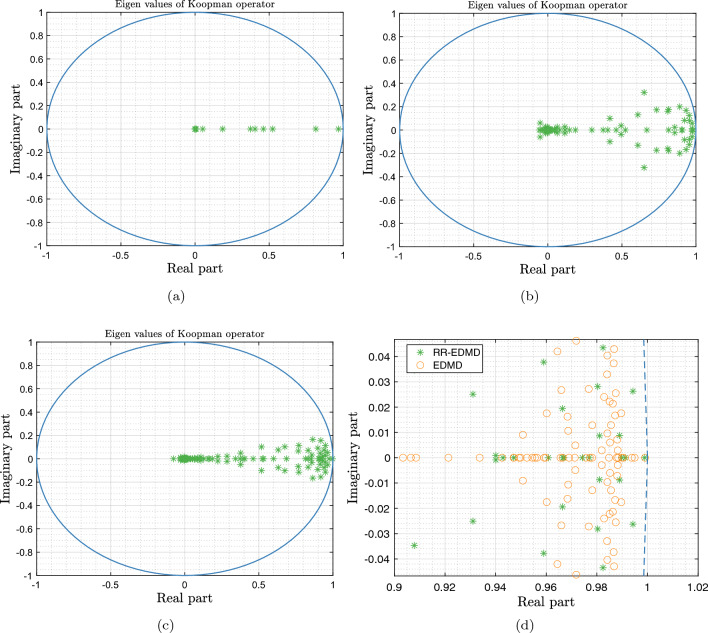
Eigenvalues of the Koopman operator after (**a**) 100 iterates; (**b**) 500 iterates; (**c**) 1000 iterates. (**d**) Dominant eigenvalues of the Koopman operator computed using RR-EDMD and dominant eigenvalues of the deterministic power network.

**Figure 9:**
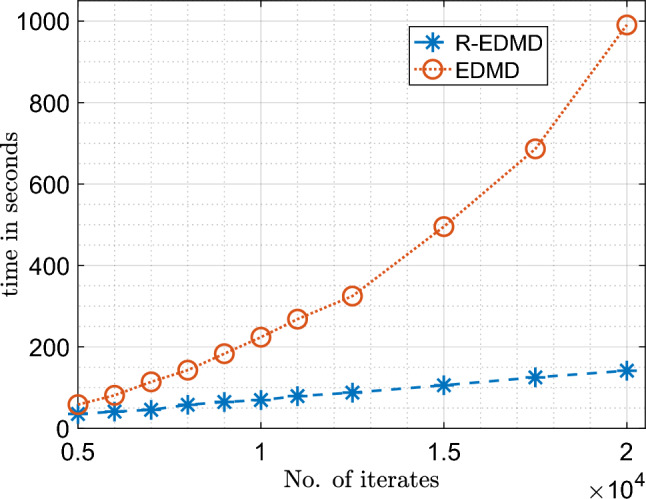
Comparison of computation time for recursive learning using normal EDMD and RR-EDMD.

Considering the initialization given in algorithm 1, a robust Koopman operator corresponding to the IEEE 68 bus system representing the underlying load changes is computed in real-time from streaming noisy PMU data. As the frequency and voltage magnitudes are considered at each bus, there is a total of 136 states and we use 150 Gaussian radial functions as observables, to compute the robust Koopman using RR-EDMD. Figure 8a–c respectively show the eigenvalues of the learnt Robust Koopman operator after 100, 500 and 1000 measurements. Moreover, we can observe that the robust Koopman updated with every new measurement yields a stable representation (all eigenvalues lying inside the unit circle). Essentially, applying the proposed RR-EDMD, the power system dynamics is learnt using measurements from a few seconds. However, the Koopman operator learning is a continuous process as the PMU data streams in and the power system properties such as stability margins can be studied at any given time-point. Furthermore, we compared the computational time of RR-EDMD against that of EDMD, shown in Fig. 9. It can be seen that the proposed RR-EDMD outperforms the standard EDMD method.

### Scalability of RR-EDMD with dimension of the underlying system

**Figure 10:**
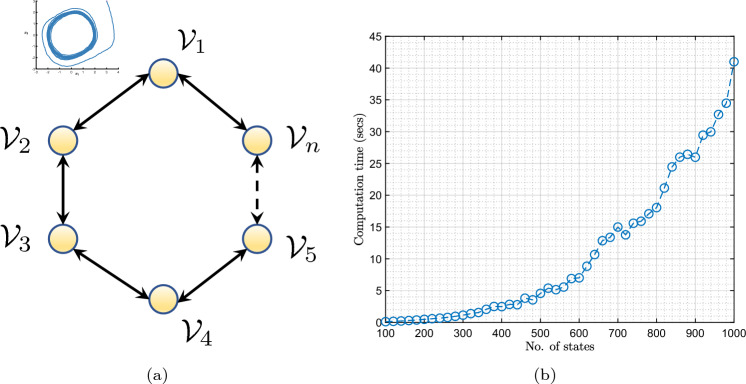
(**a**) Network of Van der Pol oscillators. (**b**) Computation time for recursive learning using RR-EDMD, as the number of states in the system increases.

In the previous two examples, we compared the computation times of the proposed RR-EDMD method and the existing EDMD method and found that RR-EDMD outperforms EDMD. In this subsection, we analyze how the scalability of the RR-EDMD algorithm as the number of states in the underlying system increases. In particular, we considered a ring network (Fig. 10a) of Van der Pol oscillators and recorded the computation times of the Koopman operator using the RR-EDMD algorithm as the number of oscillators in the system increases. The equation of motion of the *i*th oscillator is given by

$$\begin{aligned} \ddot{x}_i(t)=\mu (1-x_i^2(t)) - x_i(t) + \mathcal {L}_ix(t) + \sigma \xi _i(t), \end{aligned}$$

where $x_i(t)$ is the position variable of the *i*th oscillator, $x(t)=[x_1(t), \ldots , x_n(t)]^\top$, $\mathcal {L}_i$ is the *i*th row of the network Laplacian, $\mu \ge 0$ is the damping constant, $\sigma >0$ is a constant and $\xi _i(t)\in {\mathbb {R}}^2$ is an i.i.d. Gaussian noise of zero mean and unit variance. We varied the number of oscillators from 50 to 500, so that the number of states of the system varied from 100 to 1000. However, it is to be noted that the Koopman operator is computed in the space of the Koopman observables. In this example, we used 15 radial Gaussian basis functions per oscillator, so that the size of the Koopman operator varied from $750 \times 750$ to $7500 \times 7500$. For simulation purposes, we used the same $\mu =0.8$ and $\sigma =0.2$ for all the oscillators.

Figure 10b shows how the computation time varies with the number of states. On the face of it, it seems that the computation time varies almost quadratically with the number of states. However, it should be noted as the number of states increases, so does the number of dictionary functions, leading to the quadratic nature of the plot. However, if the number of dictionary functions remains constant, the computation time varies linearly with the number of states (Fig. 4). Since the RR-EDMD algorithm scales almost linearly with the dimension of the system, it can be used for the identification of large-dimensional systems in real time as well.

## Conclusions

In this paper, we address the important problem of learning the dynamics of a general dynamical system, from noisy measurements in real time. In particular, we use the Koopman operator framework for data-driven learning, so that one obtains a linear system representation of the underlying dynamical system. We resort to Robust Koopman operator estimation to mitigate the effect of measurement noise and we propose an iterative algorithm for recursively learning the Robust Koopman operator from streaming data. We show that the proposed framework is substantially faster than the existing EDMD algorithm, thus making it practical to learn the dynamics in an online fashion and in real time. We also demonstrate the efficiency of the proposed algorithm by applying it to identify the Van der Pol oscillator, the IEEE 68 bus system and a ring network of Van der Pol oscillators.

## Data Availability

The data for the power system is available from the corresponding author on reasonable request.

## Code availability

The codes used in this paper is available in the following address: https://drive.google.com/file/d/1FNo6SY5c3a4yoVpFYXCYqBX_OSes_TAu/view.
